# Optimizing Online Social Networks for Information Propagation

**DOI:** 10.1371/journal.pone.0096614

**Published:** 2014-05-09

**Authors:** Duan-Bing Chen, Guan-Nan Wang, An Zeng, Yan Fu, Yi-Cheng Zhang

**Affiliations:** 1 Web Sciences Center, School of Computer Science and Engineering, University of Electronic Science and Technology of China, Chengdu, China; 2 Department of Physics, University of Fribourg, Fribourg, Switzerland; Cinvestav-Merida, Mexico

## Abstract

Online users nowadays are facing serious information overload problem. In recent years, recommender systems have been widely studied to help people find relevant information. Adaptive social recommendation is one of these systems in which the connections in the online social networks are optimized for the information propagation so that users can receive interesting news or stories from their leaders. Validation of such adaptive social recommendation methods in the literature assumes uniform distribution of users' activity frequency. In this paper, our empirical analysis shows that the distribution of online users' activity is actually heterogenous. Accordingly, we propose a more realistic multi-agent model in which users' activity frequency are drawn from a power-law distribution. We find that previous social recommendation methods lead to serious delay of information propagation since many users are connected to inactive leaders. To solve this problem, we design a new similarity measure which takes into account users' activity frequencies. With this similarity measure, the average delay is significantly shortened and the recommendation accuracy is largely improved.

## Introduction

The information and communication technologies lead us to an information-rich era where recommender systems are widely used to filter out irrelevant information [1]–[3]. Recommendation algorithms include correlation-based collaborative filtering [4]–[6], Bayesian clustering [7], probabilistic latent semantic analysis [8], matrix decomposition [9], [10]. Many issues related to recommender systems have been studied, such as the diversity of recommendations [11], [12], the effect of network topology [13], and ground user [14]. Recent researches show that social influence [15] is more powerful than the purely mathematical analysis based recommendation, as people are more likely to accept the recommendations coming from their friends or peers [16]. Hence, a new technology named social recommendation has emerged [17]–[19] in which users (followers) can select some other users as information sources (leaders) and the information will automatically flow from leaders to followers. This framework has been successfully applied in many real online websites, such as *delicious.com*, *twitter.com* and *digg.com*. The information can refer to news, movies, books, bookmarks, and so on. Without losing any generality, news is used as an example in this paper. When a piece of news is submitted or approved by a user, it will be forwarded to her followers. The diffusion of news thus depends on the structure of leader-follower network, with higher transmission probability of news if users with higher similar tastes are linked [20].

Recently, an adaptive news recommendation model is proposed [21]. In this model, when a user reads news, she can either “approve” or “disapprove” it. If approved, the news will be forwarded to her followers. With the spreading of news, the leader-follower network will be updated, that is, the least suitable leader of a user will be replaced by a better one according to the quality of the leader. The quality of her leader is measured by the similarity based on their past assessments on news. The model has been extensively tested by additional aspects like users' reputation [22], implicit ratings [23], local topology optimization [24], leadership structure [25] and link reciprocity [26]. More recently, Cimini et al. [27] introduced two settings for modeling users' tastes and showed that the heterogeneous setting of users' tastes was closer to the real case than homogeneous setting.

Confirmed by many empirical analysis, it is now well-known that the activity frequency of online users are heterogenous [28]–[30]. However, in the original adaptive news recommendation model and the following studies, users are randomly selected to be active (i.e. submitting or reading news), which indicates that the activity frequency of users are set to be homogenous. In this paper, we find that the propagation of news is seriously delayed when some classic similarity metrics are applied in the heterogenous users activity setting. Moreover, the recommendation accuracy (i.e. approval fraction of the news) is lowered as well. To solve this problem, we propose a new similarity measure which takes user activity into account. The simulation shows that the propagation delay is considerably shortened and recommendation accuracy is largely improved. Finally, we introduce a more general similarity definition in which the weight on users' news assessments and users' activity is tunable. With this, the effectiveness (accuracy) and efficiency (time delay) of the information propagation is further improved.

## Materials and Models

### Empirical analysis

**Figure 1:**
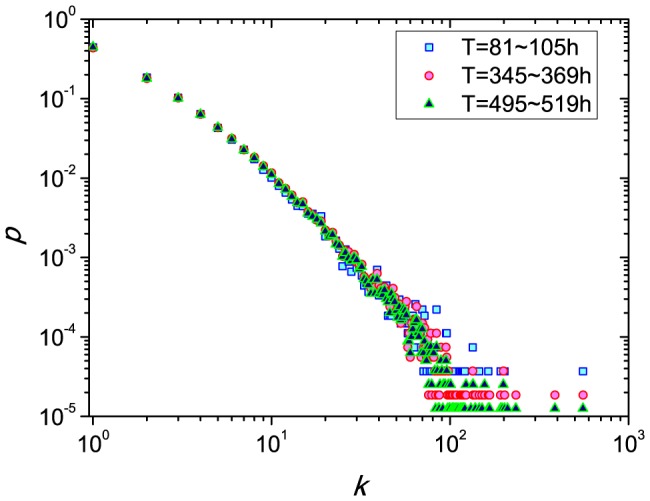
The distribution of activity frequency of users on Digg.com over a period of a month in 2009.

To begin our analysis, we study the distribution of user activity frequency in real systems. Here, we consider the dataset of digg.com [31], which contains 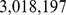 votes on 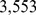 popular stories made by 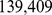 distinct users over a period of a month in 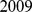. In this dataset, users and stories form a bipartite network in which a link between a user 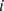 and a story 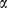 exists if user 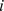 reads story 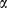. The degree of a user represents the number of stories read by her (i.e. the activity of this user). Figure 1 shows the degree distribution in three observation time windows. Clearly, the distribution follows a power-law with exponent around 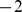. The results confirm that users are very heterogenous in the frequency of their online activity.

**Figure 2:**
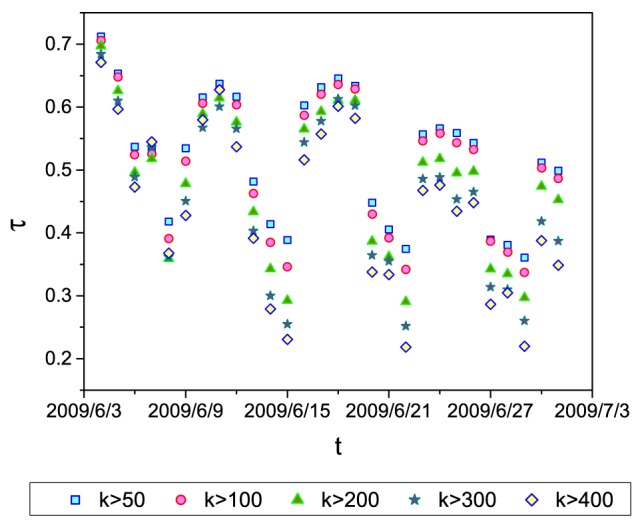
The correlation of the activity frequency of users in two adjacent days. The actually dates are marked in the horizontal axis. 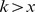 in the legend means that we only take into account the users with total activity being larger than 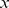.

Furthermore, we employ the Kendall's tau coefficient (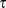) to calculate users' correlation of activity frequency in two adjacent periods. The length of the period is set as one day, so we are actually calculating the Kendall's tau coefficient of users' activity frequency in each day and the previous day. As shown in Fig. 2, 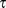 is always larger than zero, which means that users' activity frequency is positively correlated in time. Moreover, we observe that there is some periodic fluctuation in Fig. 2. With the actual dates, we check carefully the reason for this periodic fluctuation. We find that each period is one week, and the correlation is higher in weekdays than in weekends. We conjecture it is because people's live is regular in weekdays but diverse in weekends.

### Model description

In the original adaptive news recommendation model [21] and the following studies [22]–[27], users' activity frequencies are assumed to be homogenous, which is inconsistent with the results of above empirical study. To make the information propagation model closer to the real system, we introduce the heterogeneity of users' activity frequency to it. Our model will be directly built on the original news-sharing model in ref. [21]. The system consists of 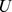 users. Each of them is connected by directed links to 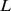 other users, who represent her news sources and to whom we refer as her *leaders*. The value of 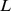 is fixed as users can follow a limited number of sources. Users receive pieces of news from their leaders, and eventually assess them. In addition, they can introduce new content to the system.

Evaluation of news 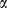 by user 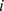 (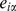) is either 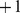 (liked), 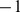 (disliked) or 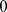 (not read yet). The set of evaluations from any pair of users 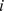 and 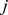 is the basis to compute their similarity of their interests (or reading tastes), which is denoted as 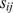. The explicit recipes to compute users' similarity are presented in the next section. Note that, apart from their evaluations, no other information about users is assumed by the model.

**Users' activity.** In each time step of the simulation, a given user is active with probability 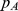. When active, a user reads the top 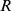 news from her recommendation list, immediately forwarding the ones she likes to her followers. In addition, with probability 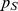 she submits a new piece of news. Different from the original model in [21], the users' activity frequency is drawn from a power-law distribution as 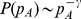 where 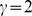.

**Propagation of news.** When news 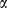 is introduced to the system by user 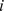 at time 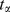, it is forwarded from 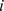 to the users 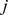 who have selected her as a leader, with a *recommendation score* proportional to their similarity 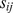. If this news is later liked by one of her followers 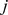, it is similarly passed further to this user's followers 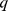, with recommendation score proportional to 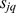, and so on. For a generic user 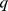 at time 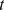, a news 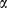 is recommended to her according to its current score: 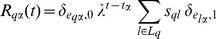
(1)where 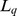 is the set of leaders of user 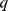. 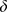 is a Dirac delta function with only two possible values: 0 and 1. If user 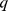 has not read news 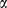, 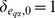 since 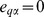 and if 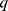 has read news 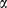, 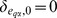 since 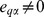. Similarly, 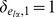 if user 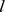 likes news 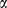, otherwise 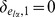. To make the fresh news fast accessed, recommendation scores are damped with time (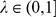 is the damping factor).

**Leader selection.** The model is adaptive. Initially, each user randomly select 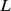 other users as her leaders. Leader-follower connections are periodically rewired to make the social network approach an optimal state where only highly similar users are connected [32]. In each rewiring, for user 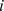, her current leader 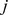 with the lowest similarity value is replaced with a new user (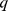) if 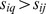. There are different selection strategies for picking new candidate leaders, which are discussed in detail in [22], [24], [26]. In this paper we employ a hybrid strategy in which the user 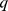 is picked at random in the network with probability 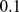, otherwise she is selected among the leaders' leaders and followers of user 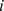 to maximize 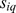. This mechanism well mimics users establishing mutual friendship relations, searching for friends among friends of friends, and having casual encounters which may lead to long-term relationships. In addition, it is an excellent compromise between computational cost and system's performance [24].

### Measure of users' similarity

An essential ingredient of the social recommendation algorithms is the estimated similarity of users' reading tastes, which regulates the news' flow over the system by determining the leaders' selection from users (i.e., the link structure of the network) and recommendation scores of news. Since only users' ratings and records of activities are known, the similarity of a pair of users has to be estimated from their assessments on news, which in our case can be either approved, disapproved, or not rated.

The first similarity measure considered is introduced in [21] as 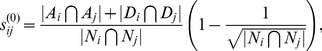
(2)where 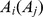 is the set of news approved by user 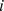 (user 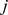), 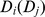 is the set of news disapproved by user 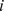 (user 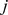), and 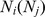 is the set of news read by user 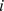 (user 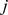). The term 
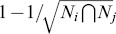
 is used to remove the effect of statistical fluctuation. If a user 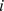 and a user 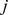 share a small number of commonly read news, they are more likely to achieve “perfect” similarity 1. After multiplying this term, the similarity measure will give this user pair a very low similarity value. In sampling of 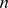 trials, the typical relative fluctuation is of the order of 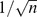. Therefore, we select the above form.

In [33], it is shown that Eq. (2) works well only in the system where tastes of users are homogeneously distributed, i.e., each user has the same number of interested fields. To achieve a more accurate leader assignment in the system where users can have different number of tastes, an asymmetric similarity measure is defined as 
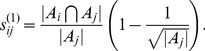
(3)



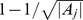
 here is also used to remove the effect of statistical fluctuation.

In this paper, we consider the systems where users' activity distribution is uneven. Some users can be extremely active and read many news, so that their followers can constantly receive fresh news. On the other hand, if one user is connected to many inactive leaders, the news received by her will be very limited. Therefore, recommending highly inactive users is meaningless. Considering this, we modified the similarity in Eq. (3) as 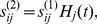
(4)where 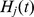 is a measurement of user 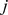
's activity. Actually, there are many other previous works showing that online users' activity frequency is unevenly distributed[34], [35].

Users' active frequencies 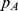 are users' inherent feature and unknown by the recommender system. We design the following way to estimate 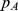 of the users. Instead of taking the whole history into account, we only use the recent record of activities within a time window 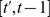 with length 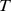 (In our simulation, 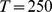 generally works best, see Supporting Information S1). The estimated probability for user 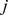 to get online is 
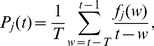
(5)where 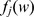 is user 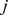
's online times from time 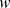 to time 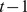. If 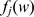 equals 0 for each 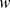, we set 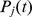 as 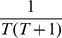. In Eq. (5), users' recent record plays a more important role. This is very useful in real systems, since the correlation of real users' activity frequency is generally high in short term (See Fig. 2).

**Figure 3:**
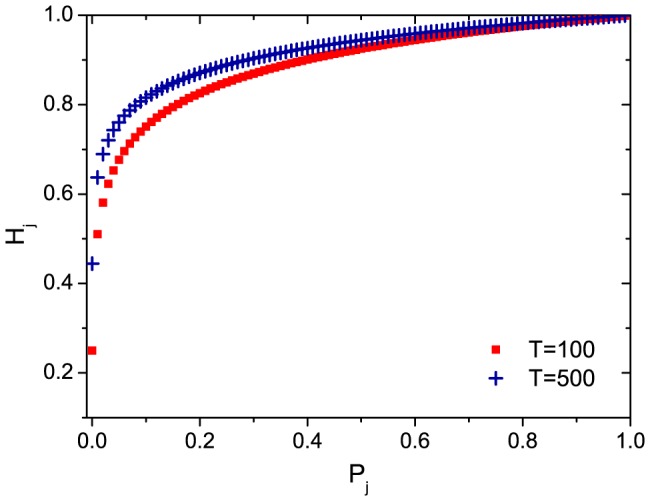
The relation between 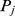 and 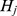.

However, 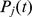 cannot be directly used as 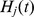 in Eq. (4). Since 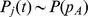, 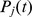 follows a power-law distribution. If it is used as 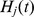, some users with high activity will dominate the similarity matrix and be always selected as the leaders of others. In order to solve this problem, we proposed a logarithmic way to embed 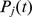 in 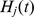. After normalization, it reads 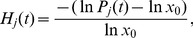
(6)where 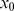 is the possible lowest value of 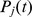, set as 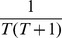. After simplification, 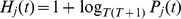. In this definition, 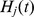 can distinguish different users and the most inactive users are punished severely. However, the majority gets 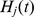 over 0.5, as shown in Fig. 3. We rewrite 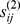 as 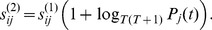
(7)


In our simulation, we actually compare our method 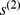 to with some start-of-the-art similarity methods based on both news-reading and topology. The results show that 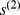 can outperform others, see Supporting Information S1. In the following, we will study the behavior of the system under these similarity metrics. For numerical tests of the model, we use an agent-based framework.

### Agent-based simulations

To model users' judgments of read news we use a vector model where tastes of user 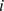 are represented by a 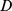-dimensional taste vector 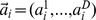 and attributes of news 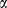 are represented by a 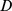-dimensional attribute vector 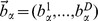. Similar vector models are often used in semantic approaches to recommendation [36]. Opinion of user 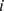 about news 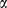 is based on the overlap of the user's tastes and the news's attributes, which can be expressed by the scalar product 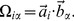
(8)


We assume that user 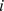 approves news 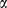 only when 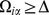, disapproves otherwise, where 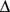 is the users' approval threshold: the higher it is, the more demanding the users are. Here, we adopt the heterogeneous setting of the taste/attribute vectors.

Each user has preference for a variable number of 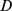 available tastes. Each taste vector has a different number of elements equal to one (active tastes, denoted as 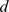) and the remaining elements are zero. In this paper, we assume 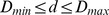 where 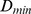 and 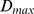 are the minimum and maximum number of active tastes that users can have, respectively. Moreover, we assume that each news' attribute vector has a fixed number 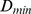 of active attributes (number of ones), which are randomly chosen among the active tastes of the user who submits it.

Simulation runs in discrete time steps. Assuming no a priori information, the starting network configuration is given by randomly assigning 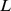 leaders to each user. Then in each simulation step, an individual user is active with probability 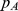. When active, the user reads and evaluates the 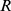 top-recommended news she has received and with probability 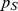 submits a new news. Connections are rewired every 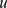 simulation steps. Parameter values used in all following simulations are given in Table 1. A detailed study of the effect of the parameters on the model can be seen in [24].

**Table 1:** List of parameters used in simulations.

parameter	symbol	value
Number of users	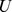	3498
Number of leaders per user	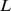	10
Dimension of taste vectors	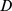	20
Minimum active elements per vector	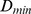	4
Maximum active elements per vector	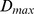	8
Users' approval threshold	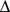	3
Index of distribution	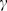	−2
Probability of submitting a news	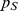	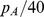
Number of news read when active	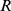	3
Damping of recommendation score	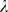	0.9
Base similarity for users	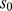	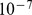
Period of the rewiring	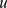	10

### Metrics

Three metrics are used to measure the performance of the recommendation models: *approval fraction* 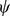, *average differences* 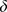, and *average delay* 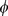.

**Approval fraction 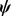.** The ratio of news' approvals to all assessments is an obviously important measure of the method's performance. This number, referred to as approval fraction, tells us how often users are satisfied with the news they get recommended. The higher the 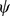 is, the more accurate the recommendation is, and users are more satisfied correspondingly. It can be defined as 
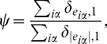
(9)where term 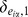 equals one if user 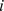 approved news 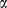 and zero otherwise, and term 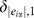 equals one if user 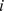 has rated news 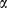 and zero otherwise.

**Average differences** 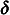. In the computer simulation, we have the luxury of knowing users' taste vectors and hence we can compute the number of differences between the taste vector of a user and the taste vectors of the user's authorities. By averaging over all users, we obtain the average number of differences. Obviously, the less are the differences, the better is the assignment of authorities. The average differences are defined as 
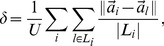
(10)where 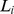 is the set of leaders of user 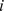, and 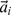 (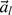) is the taste vector of user 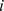 (user 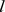).

**Average delay 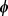.** The freshness of the news is very important. Once the news becomes old, it is of no interest to users. The average delay measures the novelty of the news read by users. A small average delay indicates that users are always reading fresh news. The average delay is defined as 
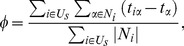
(11)where 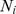 is the set of news read by user 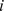, 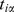 is the time when 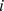 reads news 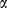, 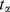 is the submitted time of news 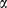, and 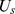 is set of users. The smaller the 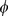 is, the news read by users will be fresher.

## Results and Discussion

We now study the described adaptive social recommender system under different definitions of the similarity measure employed. For comparison, initial conditions and parameters for all simulations are identical, as listed in Table 1. We obtained the average differences, approval fraction and average delay resulting from each similarity definition. We first consider the case where the user activity and the number of user interest are uncorrelated and the results are shown in the uncorrelated case in Table 2. As expected, 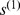 enjoys a higher approval fraction and a smaller average difference than 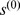. The results are consistent with ref. [27], [33]. However, 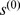 results in a smaller average delay than 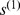. Among these methods, 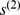 performs the best in all three metrics. These results suggest that introducing the users' activity to the similarity measure can significantly speed up the propagation of news in online systems so that users mostly receive fresh news. Moreover, it improves the leader assignment, resulting in a more accurate recommendation of news for users.

**Table 2:** Three evaluation metrics on different similarity measures.

	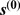			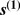			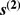		
	Pos.	Neg.	Uncorr.	Pos.	Neg.	Uncorr.	Pos.	Neg.	Uncorr.
Average delay 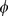	247.08	426.52	1058.10	377.23	881.12	1383.01	103.93	109.30	123.53
Average differences 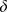	6.1838	7.5234	8.3693	5.7045	6.7641	6.9015	5.5779	5.5033	5.3161
Approval fraction 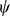	0.4561	0.3034	0.2280	0.3873	0.3324	0.3198	0.4274	0.4187	0.3502

One concern of the new similarity measure is that the network updating algorithm might focus too strongly on the activity frequency of leaders rather than the taste overlap of the leader and follower, putting the information recommendation accuracy at risk. Accordingly, we further introduce a parameter 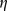 to adjust the effect of users' activity in the 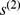 similarity calculation: 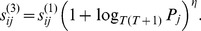
(12)


Parameter 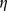 controls the weight assigned to 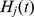. When 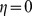, Eq. (12) reduces to 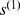, and when 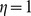, Eq. (12) reduces to 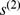.

**Figure 4:**
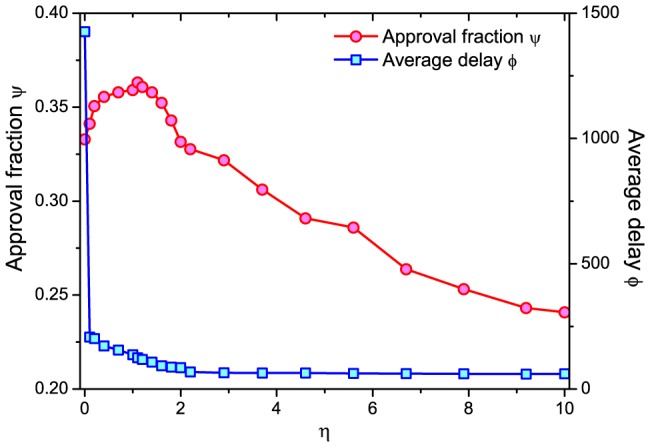
Stationary values (simulation step 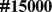) of *average delay* 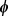 and *approval fraction* 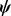 in the adaptive system ruled by 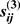, and for different values of the parameter 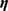.

The stationary values of average delay 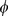 and approval fraction 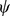 obtained by using 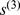 under different 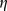 are reported in Fig. 4. One can immediately see that there is a maximum approval fraction when adjusting 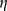. On the other hand, the average delay drop dramatically once 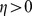 and then decreases monotonously with 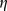. Compared to the case where the users' activity is not considered in the similarity calculation (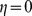), the approve fraction can be improved and average delay can be considerably shortened. Interestingly, the optimal 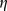 is around 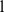, corresponding to the case of 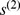.

We further consider the situation where the user activity and the number of user interest are correlated. Three cases are considered in this paper: positive correlation, negative correlation, and no correlation. We first compare three evaluation metrics under different correlation settings (see Table 2). One can immediately see that both positive and negative correlation between the user activity and the number of user interests can significantly shorten the average delay 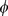 of the news. However, the delay from 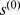 and 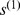 are still longer than 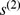. The advantage of 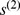 can also be found in the average difference 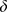. A lower average difference indicates that the network is adapted to a better state for news propagation. We can also see that 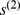 enjoys the highest approval fraction 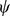 in almost all cases. When the correlation between the user activity and the number of user interests is positive, the approval fraction of 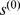 is a bit higher than that of 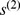. However, the delay in 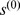 in this case is more than twice longer than that of 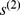. Taken together, 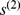 is a very effective and robust similarity measure for recommending leaders in online social systems.

**Figure 5:**
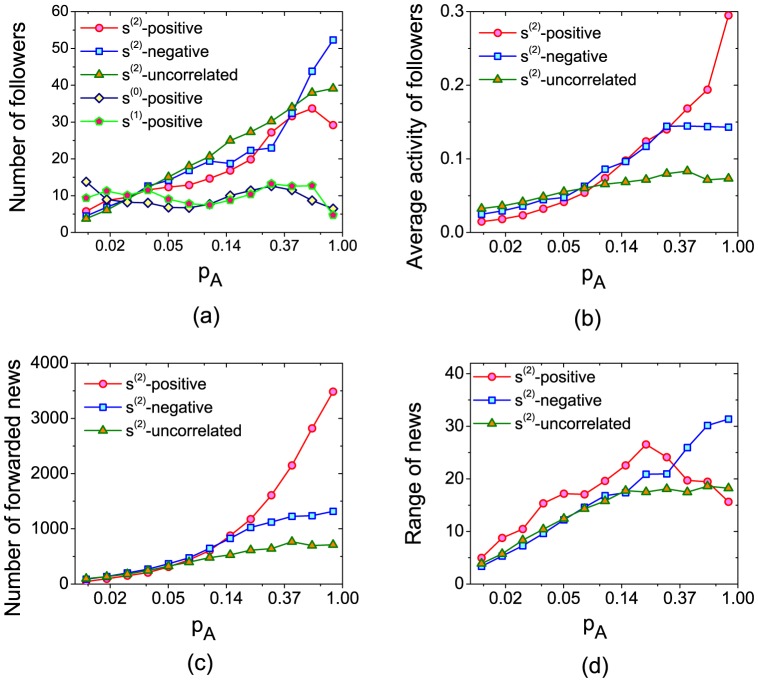
The (a) number of followers, (b) average activity of followers, (c)number of forwarded news and (d) spreading range of users with different activities (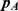).

The leader-follower networks after the systems reach stable state is studied. We first investigate the in-degree distribution of nodes (i.e. the distribution of number of followers). The results show that the largest in-degree in 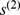 is smaller than that in 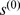 and 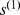. This is because users in 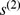 select leaders according to not only the similarity but also the activity frequency. It is more difficult for the largest in-degree nodes in 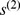 to attract as many followers as in 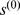 since these users have both high similarity to others and high activity frequency. We then study some properties of the users of different activities in Fig. 5. Fig. 5(a) shows the relation between user activity and the number of her followers. As discussed above, if the leaders of a user are with low activity, the user may have no news to read and the propagation of news will be largely delayed. This happens a lot in the original similarity measure 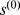 and 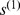 (See the flat curves of them in Fig. 5(a)). We didn't plot the curves of negative and uncorrelated cases in 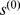 and 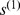 because they are as flat as in the positive correlation case. In 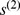, the users with higher activity frequency have more followers, which makes users with rich number of news to read. Moreover, we observe that the users with higher activity and fewer interests (see the negative correlation case when 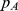 is large) have more followers. In ref. [33], it is already pointed out that the users with few interests are good information resource and should be selected as leaders (since they are specialized in their fields). As shown in Fig. 5(a), 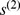 recommends the users with high activity and few interests as leaders to others. This again supports that 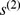 is a good similarity measure.

In Fig. 5(b), we present the relation between users' activity and the average activity of their followers. We can see that the users with higher activity and wider interests (i.e. large 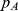 in positive correlation) have more active followers. Generally, the interests of the followers are wider than that of the leaders [33]. The followers of large 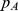 users will have wide interests and thus high activity.

Moreover, it is interesting to identify which kind of users can be the information hubs in online social networks. We first investigate the number of forwarded news of different users. As shown in Fig. 5(c), the users with higher activity and wider interests forward more news. With wider interests, these users are more likely to approve the news from their leaders, which results in a large number of forwarded news from them.

However, the results in Fig. 5(d) indicate that the users with high activity and wide interests are actually not information hubs. We report the spreading range when the news is originated from different users in Fig. 5(d). The spreading range here is defined as the number of users who finally read the news. As discussed above, the users with fewer interests are more specialized in their fields and their followers are more likely to approve the news from them. Therefore, the news originated from users with higher activity and fewer interests will spread wider. The results imply that the active and specialized users are the information hubs in online social networks.

## Conclusion

In this paper, we study a new multi-agent based model for information propagation and recommendation on online social network. The original online information propagation model was proposed in ref. [21] where users' activity frequency is assumed to be homogeneously distributed. Since the empirical study of the online news-sharing systems suggests that users' activity frequency distribution actually follows a power-law distribution, we introduce the heterogeneity to users' activity frequency distribution to the model in ref. [21]. We find that previous similarity methods for leader recommendation connects many users to inactive leaders, resulting serious delay of information propagation and low approval fraction of news.

To solve this problem, we propose a new similarity measure which takes users' activity frequency into account. With the new similarity measure, the suitability of a leader is evaluated according to not only the similarity but also the activity frequency. The numerical simulation shows that our method can outperform the existing ones in network optimization for information recommendation, in both approval fraction and information delay. Finally, we introduce a parameter to adjust the effect of users' activity in the similarity calculation. We find that the leader recommendation can be further improved by this parameter.

Since real online users have heterogenous activity frequency, we believe that our method will be very useful from practical point of view. Since real online news-sharing systems can be different from current model in parameter settings or even news propagation mechanism, the optimal weight of users' activity frequency in the similarity calculation should be determined by some preliminary testings. One possible way is to implement the method first on a small subset of users. After obtaining the optimal balance between users' activity frequency and similarity from the learning procedure, the method can be applied to the whole systems.

## Supporting Information

Supporting Information S1 **Supporting text and figures.**
(RAR)
